# Phytoliths reveal the earliest fine reedy textile in China at the Tianluoshan site

**DOI:** 10.1038/srep18664

**Published:** 2016-01-14

**Authors:** Jianping Zhang, Houyuan Lu, Guoping Sun, Rowan Flad, Naiqin Wu, Xiujia Huan, Keyang He, Yonglei Wang

**Affiliations:** 1 Key Laboratory of Cenozoic Geology and Environment, Institute of Geology and Geophysics, Chinese Academy of Sciences, 100029, Beijing, China; 2 Center for Excellence in Tibetan Plateau Earth Science, Chinese Academy of Sciences, 100101, Beijing, China; 3 Department of Anthropology, Harvard University, 02138, Cambridge, USA; 4 Zhejiang Provincial Institute of Cultural Relics and Archaeology, 310014, Hangzhou, China

## Abstract

Textiles are among the longest and most widespread technologies in human history, although poor preservation of perishable artifacts in Paleolithic and Neolithic contexts makes them difficult to unearth and has hampered study of their production and use. Here we report evidence of a plain-woven mat from the Tianluoshan site, Zhejiang, Eastern China. Phytolith and AMS dating from the mat and modern reference collections shown that the mat was made of reeds (*Phragmites australis* (Cav.)) and dated to 6775–6645 cal. yr. BP. This is the earliest directly dated fiber artifact so far known in China, over at least one thousand years earlier than any established dates for woven remains elsewhere. The evidence of the mat and other related remains suggest that textile products might occur earlier than 7000–8000 years ago and are significant for understanding the history of textiles, as well as production and human adaptation in Neolithic China.

Textiles, webs of interlaced threads produced on a loom or frame with continuous plane surfaces, are recognized as a significant technology in human evolution^1^,^2^,^3^. The production of baskets, mats, and clothes, all constructed of materials available to early societies, boosted the human capacity for adaptation^1^,^4^. Specialist analysis of weaving and the materials used for early textiles greatly contributes to our knowledge of early society and permits an expanded understanding of the range of technological activities that can be considered as the subject of anthropological inquiry^1^,^3^,^5^,^6^. Since an understanding of technological development is fundamental to any attempt to evaluate the development of culture and society, the origin of textile production is among the critical foci in world archaeology^1^,^7^,^8^,^9^,^10^.

In East Asia, textile crafts, one of the domains of technological practice with the longest continuous developmental history from ancient times to the present without changes in basic technique, are recognized as one of the symbols of Chinese civilization^11^. Evidence to date suggests the fiber of wild plants was the principal raw material for early textiles and cordage^1^,^12^, as was true elsewhere in the world^13^. However, in contrast to our understanding of plant use in foods, far less is known about the history of plants used as or in artifacts^14^,^15^,^16^. When, who and how people first made textiles is poorly known due to the lack of surviving fragments identified from ancient contexts^17^,^18^. Furthermore, because the most common evidence of textiles is limited to imprints of the weave on fragments of pottery^1^,^18^,^19^,^20^, identification of raw materials or specific functions beyond cordage used for ceramic production can be inaccurate or inadequate^1^,^3^,^15^,^16^,^17^,^21^.

Perishable prehistoric mat-like residues have recently been found in Far Eastern, Russia dated *ca*. 9000–8000 cal. yr. BP., however, the forms of the objects and related woven techniques, which are crucial criteria for the definition as textile, need to be further discussed^12^. Early mat-like crafts have also been found in middle Neolithic China, at the sites of Hemudu and Tianluoshan sites, but to date these only have been described briefly in the excavation reports, and previously none have had the raw material used identified to a species-level, nor have they been directly dated 22,23,24.

Tianluoshan (30° 01′ 27″ N, 121° 22′46″ E), a Neolithic settlement of the Hemudu Neolithic culture, is located in Yuyao city, Zhejiang Province, two to three meters above present-day sea level^23^. Excavations between 2004 and 2007 revealed preserved wooden posts, boat paddles, wooden and bone tools, pottery and ground-stone axes characteristic of the Hemudu culture, and animal and fish remains, as well as well-preserved plant remains. Pieces of prehistoric mat-like remnants were found beneath a floodplain sediment layer that was about 3 meters thick^23^. Radiocarbon (14C) measurements date its occupation to around 7000 to 6000 yr. cal. BP^25^.

Here, we present phytoliths, starch, and radiocarbon dating from the woven objects at the Tianluoshan site (Fig. 1). Modern reference collections are used to identify the associated raw materials. Our study reveals that the woven material belongs to reed matting dating to as early as 7000 yr. cal. BP, and indicates it is the earliest fine textile mat in China, predating known evidence of silk or any other fiber products.

## Results

**Figure 1:**
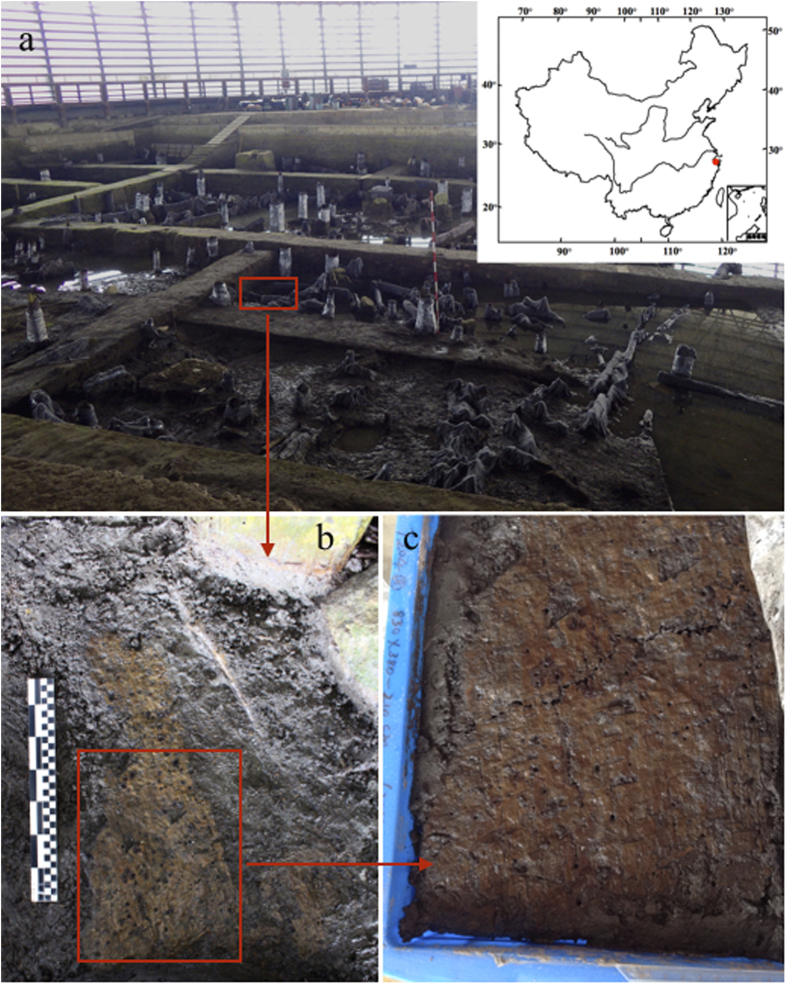
Maps of representative early reedy mats found in China. (**a**) the red rectangle shows the location of the mat residue in the Tianluoshan site; (**b**) the condition when the mat was unearthed; (**c**) sampling kit used on the mat. The map in this figure was generated by Microsoft PowerPoint 2011 and CorelDraw 11.

**Figure 2:**
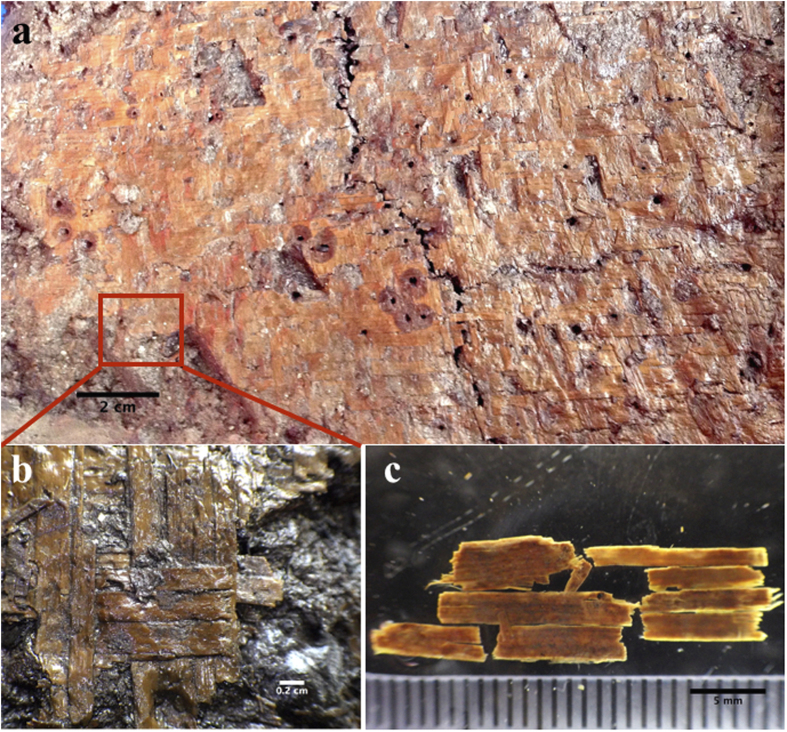
The mat unearthed from the Tianluoshan site. (**a**) overview of the mat; (**b**) enlarged view of the mat; (**c**) the dated material from the mat.

The yellow colored mat-like material was found horizontally embedded in black mud, 210 cm in depth in the excavation unit T204, in the 8^th^ stratum, which belongs to the first stage of the Tianluoshan site (Figs 1,2)^23^. The irregularly shaped object is about 50 cm in length, 20–40 cm in width, and was found within the living and working zone of the ancient community (Fig. 1). The delicate object was about 0.2 cm in thickness. Every strip of the mat was nearly the same width of *ca*. 1 cm, and composed of four to six elements of *ca*. 0.2 cm each (Fig. 2).

Boxes measuring 40 × 50 cm were used to sample and preserve the artifact. A piece of plant material collected from one corner of the object was AMS dated at Beta analytic Inc. (Fig. 2c). The sample was dated between 6775–6645 cal. yr. BP in two-sigma calibration, in accordance with the well-known Hemudu culture and Tianluoshan occupation^22^,^23^,^25^.

**Figure 3:**
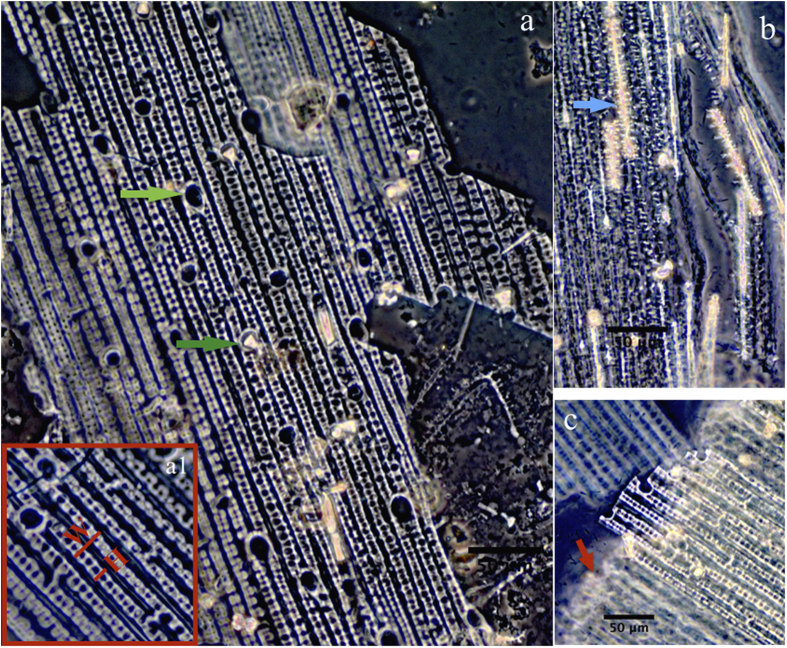
Phytolith skeleton from the mat. (**a**) n-undulating pattern of outer epidermal layer with rondel (dark green arrow) and hair cell phytoliths (light green arrow), (a1) definition of the parameters; (**b**) undulating patterns occurred with adnate silicified extracellular sheet (keratose layer) and silicified dendritic phytolith (blue arrow); (**c**) undulating patterns occurred with hypoderm fibers (red arrow).

A 2 × 2 cm piece was collected from the same corner of the object for phytolith diagnosis of raw materials. Based on our observations and statistics of structures of undulating patterns, we found that three parameters can be used to characterize the morphological variations of structures of silicified cells: H = undulation amplitude of epidermal undulating patterns, W = width of undulating patterns and especially R = H/W (Fig. 3a1).

Figure 3 shows three types of phytoliths presented in the archaeological samples. The dominant type is an n-shaped undulating pattern of the outer epidermal layer with rondels and hair cell phytoliths. The three measurements of these features are as follows: H = 7.04 ± 1.32 (standard deviation) μm, W = 19.39 ± 1.86 μm and R = 0.36 ± 0.06 (N = 109) (Fig. 3a). Occasionally we find undulating patterns that occurred with adnate silicified extracellular sheet (keratose layer), silicified dendritic phytoliths (featured as extremely slim bodies with branches) (Fig. 3b) and hypoderm fibers (Fig. 3c).

**Figure 4:**
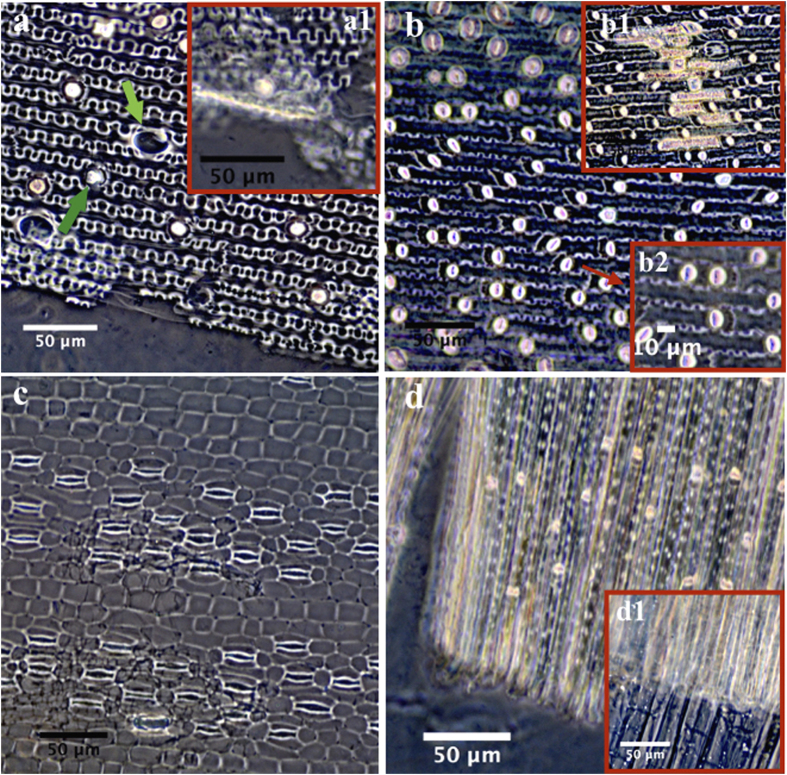
Phytolith skeleton from modern reference. (**a**) n-undulating pattern of outer epidermal layer with rondel (dark green arrow) and hair cell phytoliths (light green arrow) from a reed, (a1) silicified dendritic phytolith of reed; (**b**) undulated patterns occurred with silicified dendritic phytoliths (b1) and rondel (b2) of bamboo (blue arrow); (**c**) silicified spongy mesophyll and stomata of *Typha orientalis*; (**d**) and (d1) rondel, hypoderm fiber, and spongy mesophyll of rice.

Modern reference samples show that reed stems present the most similar kinds of phytolith morphology and also have similar measurements. They have n-shaped undulating patterns on the outer epidermal layer with rondels and hair cell phytoliths, and silicified dendritic phytoliths. Measurements of modern samples are as follows for the sample analyzed (N = 111): H = 8.09 ± 1.39 μm, W = 22.18 ± 2.60 μm, R = 0.37 ± 0.05 (Fig. 4a,a1). In contrast, Fig. 4b shows that the phytolith pattern for bamboo stems has a weaker and slightly undulating wave that is reflected by the measurement perimeters: H = 3.57 ± 0.72 μm, W = 15.80 ± 1.47 μm, R = 0.23 ± 0.04 (N = 109), and the bodies of dendritic phytoliths are wider with short and indistinct branches as well (Fig. 4b,b1). Rondels are also present in abundance in bamboo, but featured obvious ridgy lines that occurred in the middle of the rondels, which can be distinguished from those in reeds (Fig. 4b2).

Similarly, the observed undulating pattern is absented in *Typha orientalis*, which only has silicified spongy mesophyll and stomata (Fig. 4c). In rice stems, simply only rondels, hypoderm fibers, and spongy mesophylls are present (Fig. 4d,d1).

**Figure 5:**
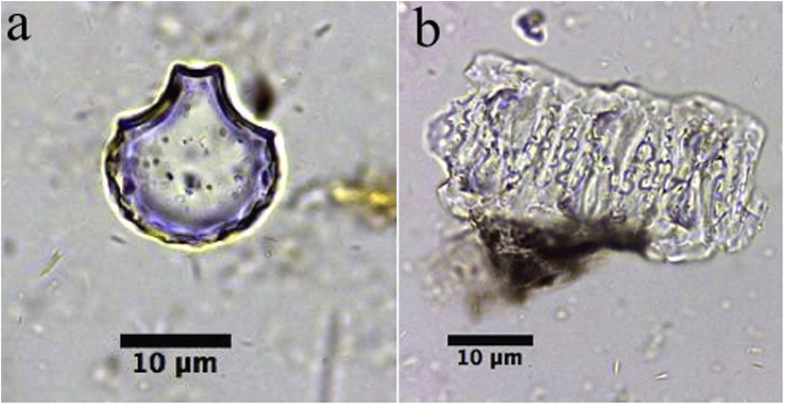
Rice phytoliths from the surface of the mat. (**a**) bulliform and (**b**) double peaked phytolith from rice leaves/stems and husk, respectively.

Attachments on the surface of the artifact were analyzed according to the references of phytolith and starch taxa in China^26^,^27^,^28^,^29^,^30^,^31^. In total, only one double-peaked and two bulliforms produced by rice were identified among 400 phytoliths (Fig. 5). The rest are common grass phytoliths. No starch grains were found.

## Discussion

Owing to the irregular shape of the artifact and its lack of edges (*ca*. 50 cm in length, 20–40 cm in width), it is difficult to determine what the ultimate form of the object was, thus phytoliths and starch grains from the surface of the fragments were analyzed. As many researchers have argued previously, woven technology constructed during the early Holocene often has a close relationship with settled communities and agriculture, as well as specialized food processing, as often basketry was used for collecting and processing seeds^32^,^33^,^34^,^35^. It is reasonable to assume that if the remains were parts of one or more containers, many crop husk phytoliths and starch grains would be found. However, the results show that only three rice phytoliths (one double-peaked and two bulliforms) were found among the 400 phytoliths, while the rest were common types from grasses, and no starch grains were found. When these data are considered together with the flat position in which the object was found when it was unearthed and the continuous plane surface, we speculate it was probably a mat fragment.

A vast variety of plant material is used in the production of woven items around the world^36^,^37^. Although the woven impressions and physical items found in many early Neolithic sites were sometimes clearly made of plant rather than animal fibers, plant identification of these artifacts to date has mainly relied on the observations of the general appearance of the materials, and explicit identification of the species used is rare owing to a lack of proper methods of identification for those easily degraded fibers^1^,^38^. Morphometric analysis of silicified epidermal cells can be used to distinguish among grasses because of specific morphological characteristics 37,39,40,41,42, and between domesticated and wild species^32^,^41^,^43^,^44^,^45^,^46^,^47^. In East Asia, three identified plant species; bamboo (Bambuseae), reed (*Phragmites australis*), and bulrush (*Typha orientalis*) were traditionally used as the preferred sources of matting, and are still used today^48^. To determine the taxa of the raw material of the mat present at the site, we paid particular attention to these three plants, and added rice (*Oryza sativa*) as well, owning to the high occurrence of rice remains in the Tianluoshan site, although rice is not commonly used for woven materials due to the relatively lower resilience of rice stems.

By comparing silicified epidermal long cells among *Phragmites australis,* Bambuseae, *Typha orientalis* and *Oryza sativa,* we revealed that reed (*Phragmites australis*) phytoliths were distinguishable, especially from Bambuseae, by their phytolith patterning and morphology. Comparing with Bambuseae, reeds feature a wider and larger n-undulating pattern and a higher R value, which can be used as a robust index to distinguish the two taxa. Furthermore, we provide a comparison of macro features (Fig. S1) that shows the surface structure of the epidermal layer and fibers from the archaeological sample (Fig. S1-a, a1) resembles those from reeds (Fig. S1-b, b1), but clearly is more slender than those from bamboo (Fig. S1-c, c1). Thus, although further systematic investigations are still needed to confirm whether the shape of epidermal long cells from reeds are exclusive, considering limited species that traditionally are used for the production of mats and baskets, we provide substantial evidence of reeds as raw materials for mats at this site based both on macro and micro features of epidermal layer (Fig. S1) and rondels, hair cell phytoliths, dendritic phytoliths and silicified epidermal long cells (Figs 3 and 4), respectively.

Unlike textile crafts found elsewhere in Asia during the early Holocene, such as those in the Near East and Japan, which were made from grasses with rigid and rough surfaces^12^,^49^, the mat found at Tianluoshan was made by fine plain weaving with tightly arranged slim strips that appear to be of equal width (*ca*. 0.2 cm) and form an extremely smooth surface (Figs 1,2). The fineness, tightness and uniformity of the mats cause us to speculate that some types of looms or frames may have been employed during production. The large quantity of split reed stems, a close set for warp and waft strips, would also be difficult to manipulate or maintain by hand without some type of controlling device^50^. We assume a ground frame or a loom would have been used. Plenty of weaving-related remains, including spindle whorls, weights, needles and cordage, were unearthed from both Hemudu (7 km to the south) and Tianluoshan and support this speculation^22^,^23^. Considering one of the features used to differentiate between basketry and textiles, which is whether the item was made with some variety of hanging or horizontal frame or loom^3^,^51^, we suggest that the mat is more close to a textile than basketry in terms of taxonomy, although basketry could be seen as a sub-category of textile with regard to woven techniques^3^.

Throughout the world, direct traces of textiles in early periods are rarely encountered. Evidence of woven products mostly occurs as impressions on clay or rare cordage and net fragments which date as far back as some 30,000 yr. BP^12^,^19^,^38^,^52^,^53^. The occurrence of fine textile, especially mats, in the New World occurs much later, starting around the beginning of the Holocene 50,51,54,55,56,57, however, this is still thousands of years earlier than any of textiles in China. The humid monsoon climate in East Asia, which resulted in the easy degradation of fibers, might be one of the reasons for the late records of textiles in China^58^. Another reason, however, may relate to the origin of agriculture.

Woven crafts feature in several ritual practices as part of specialized food-processing techniques, especially those dealing with agricultural products^33^,^34^,^35^. As we know, many pieces of archaeobotanical evidence support the view that the process of crop domestication occurred both in the Lower Yangtze and Yellow River regions during the Early to middle Holocene^59^,^60^,^61^,^62^. Moreover, looms, needles, spindle whorls, weights, cordage, and weaving impressions on ceramics have been unearthed increasingly from several early Holocene archaeological sites including Peiligang, Cishan, Banpo, Dadiwan and Hemudu^22^,^63^,^64^,^65^,^66^. Based on the common occurrence of mats in the 8^th^ layer of Tianluoshan site (about 27 pieces of mats, the largest measuring approximately 74 × 62 cm), we propose that the occurrence of textiles in China might accompany the origin of agriculture, which is to say that textiles in China might be invented earlier than currently evident in the archaeological record–earlier than 7000–8000 yr. BP. Verification of this will require further evidence. Woven aircrafts found in Kuahuqiao site may shed light on this, although direct dating and specific identification of raw materials are lacking^67^. Currently, the fine woven mat reported here is the earliest physical evidence in China of woven aircrafts, or textiles in general, over two thousand years earlier than the silk textile from the Qianshanyang site^24^ and one thousand years earlier than wild Kudzu fabric products at the Caoxieshan site^68^.

## Methods

We collected one sample from the mat-like material for raw material identification, one sample of surface attachments from both sides of the remains for phytolith and starch identification, and four modern species of reed (*Phragmites australis*), cattail (*Typha orientalis*), bamboo (Bambuseae) and rice (*Oryza sativa*) as modern reference samples. Identification was aided by the use of reference materials^5^,^6^,^7^,^8^,^9^.

The protocol used for plant phytolith analysis is the technique described by Lu *et al.* (2009) with slight modifications^43^: (1) The sample was cleaned with distilled water in a water bath to remove adhering particles; (2) Then placed in 20 ml of saturated nitric acid for over two to 12 hours to oxidize organic materials completely according to the hardness of the plant; (3) The solutions were centrifuged at 2000 r.p.m. for 5 min, decanted and rinsed twice with distilled water, and then rinsed with 95% ethanol until the supernatants were clear; (4) The phytolith sediments were transferred to storage vials. The residual subsamples were mounted onto microscopic slides in Canada Balsam medium for photomicrography and in liquid medium for counting, measuring, and line drawing; (5) Light photomicrography (phase-contrast, and microscopic interferometer) at 400 × magnification was used to determine their anatomy and silicon structure patterns.

The protocol for the recovery of starch grains from the sample follows the technique described by Yang *et al.* (2009)^69^: (1) A solution of 6% H_2_O_2_ was used to break down some of the larger charred particles by oxidation, releasing starch grains possibly trapped within them or adhering to them, as well as to oxidize some of the extraneous organic material from the residues; (2) A heavy liquid solution of CsCl at a density of 1.3 was added to the residue to remove any material with a specific gravity of less than 1.3; (3) Starch grains were floated using a CsCl solution at a density of 1.8, after which the heavy liquid was rinsed from the samples; (4) The material recovered from the second extraction was mounted in 10% glycerine and 90% water on a clean glass slide.

## Additional Information

**How to cite this article**: Zhang, J. *et al.* Phytoliths reveal the earliest fine reedy textile in China at the Tianluoshan site. *Sci. Rep.* **5**, 18664; doi: 10.1038/srep18664 (2015).

## Supplementary Material

Supplementary Information
