# Supplementary material for: Phytoliths reveal the earliest fine reedy textile in China at the Tianluoshan site
Source: Sci Rep. 2016 Jan 14;6:18664. doi: 10.1038/srep18664 (PMC4725870; doi:10.1038/srep18664)
Supplement: Supplementary Information [file srep18664-s1.pdf]

Phytoliths reveal the earliest fine reedy textile in China at the Tianluoshan site

Jianping Zhang[*1,2,3], Houyuan Lu[1,2], Guoping Sun[4], Rowan Flad[3], Naiqin Wu[1], Xiujia Huan[1], Keyang He[1], Yonglei Wang[4]

[1] Key Laboratory of Cenozoic Geology and Environment, Institute of Geology and Geophysics, Chinese Academy of Sciences, 100029, Beijing, China, [2] Center for Excellence in Tibetan Plateau Earth Science, Chinese Academy of Sciences, 100101, Beijing, China, [3] Department of Anthropology, Harvard University, 02138, Cambridge, USA, [4] Zhejiang Provincial Institute of Cultural Relics and Archaeology, 310014, Hangzhou, China.

*Corresponding author
E-mail: jpzhang@mail.iggcas.ac.cn
Tel: +1 617 460 3120
Fax: +86 10 6201 0846
Postal address: No. 19, Beitucheng Western Road, Chaoyang District, 100029, Beijing, China

This Supplementary Text file includes:
Fig. S1

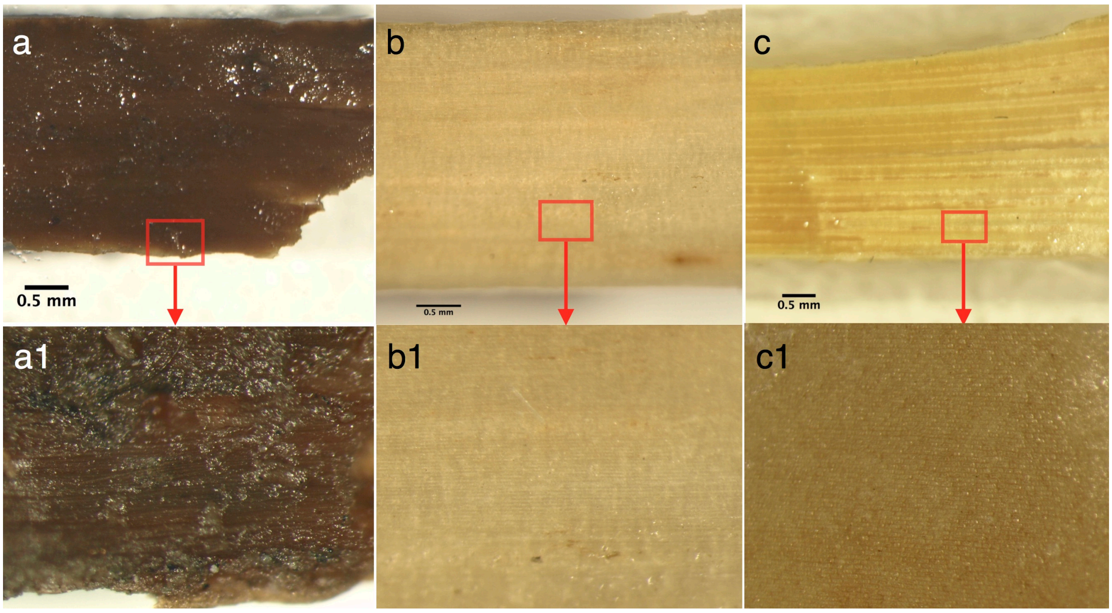

Fig. S1 The comparison of macro characters from (a, a1) archaeological remains, (b, b1) reed (*Phragmites australis*), and (c, c1) bamboo (Bambuseae).
